# Pleiotropic Functions of the Chromodomain-Containing Protein Hat-trick During Oogenesis in *Drosophila melanogaster*

**DOI:** 10.1534/g3.117.300526

**Published:** 2018-01-24

**Authors:** Ankita Singh, Debdeep Dutta, Maimuna Sali Paul, Dipti Verma, Mousumi Mutsuddi, Ashim Mukherjee

**Affiliations:** Department of Molecular and Human Genetics, Institute of Science, Banaras Hindu University, Varanasi 221005, Uttar Pradesh, India

**Keywords:** chromodomain, *hat-trick*, Gurken, oogenesis, germline mosaic, double-strand break

## Abstract

Chromatin-remodeling proteins have a profound role in the transcriptional regulation of gene expression during development. Here, we have shown that the chromodomain-containing protein Hat-trick is predominantly expressed within the oocyte nucleus, specifically within the heterochromatinized karyosome, and that a mild expression is observed in follicle cells. Colocalization of Hat-trick with Heterochromatin Protein 1 and synaptonemal complex component C(3)G along with the diffused karyosome after *hat-trick* downregulation shows the role of this protein in heterochromatin clustering and karyosome maintenance. Germline mosaic analysis reveals that *hat-trick* is required for maintaining the dorso-ventral patterning of eggs by regulating the expression of Gurken. The increased incidence of double-strand breaks (DSBs), delayed DSB repair, defects in karyosome formation, altered Vasa mobility, and, consequently, misexpression and altered localization of Gurken in *hat-trick* mutant egg chambers clearly suggest a putative involvement of Hat-trick in the early stages of oogenesis. In addition, based on phenotypic observations in *hat-trick* mutant egg chambers, we speculate a substantial role of *hat-trick* in cystoblast proliferation, oocyte determination, nurse cell endoreplication, germ cell positioning, cyst encapsulation, and nurse cell migration. Our results demonstrate that *hat-trick* has profound pleiotropic functions during oogenesis in *Drosophila melanogaster*.

The chromodomain (chromatin organization modifier) is an evolutionarily conserved motif of 40–50 amino acid residues found in a variety of proteins involved in chromatin remodeling and transcriptional regulation of gene expression in eukaryotes during development (Eissenberg 2001; Cavalli and Paro 1998). This motif was originally identified in *Drosophila* Polycomb (Pc) protein and Heterochromatin Protein 1 (HP1); both of these proteins act as repressors during heterochromatinization (Paro and Hogness 1991). Chromodomain-containing proteins are primarily nuclear proteins and bind to methylated lysines in the tail region of histone H3 through the same domain. Functional analyses have demonstrated that the chromodomain also facilitates chromatin interactions by binding directly to DNA or RNA other than methylated histone H3 (Bouazoune *et al.* 2002; Akhtar *et al.* 2000; Brehm *et al.* 2000; Kim *et al.* 2006).

Although detailed genetic, molecular, and structural studies on various *Drosophila* chromodomain proteins such as HP1 and Pc have been carried out over the past decade, there is very limited understanding about the chromodomain protein, CG34422. It has recently been renamed as Hat-trick (Htk) for its putative role in heterochromatin association and its influence on TDP-43-mediated age-dependent neurodegeneration in amyotrophic lateral sclerosis (ALS) (Sreedharan *et al.* 2015). Htk is a putative DNA-binding and chromatin-modeling protein, and its functional role in development remains to be unraveled. The gene *htk* encodes two annotated transcript variants, which are translated into two polypeptides of 186 and 259 kDa. The protein Htk harbors an AT-rich interacting domain (ARID), a chromodomain, a Retinoblastoma Binding Protein 1 N-terminal (RBB1NT/NUC162) domain, and a Tudor-Knot domain. Proteins containing these domains exhibit a range of cellular functions, which include chromatin remodeling, regulation of gene expression during growth, differentiation, and development. Moreover, mammalian orthologs of Htk, ARID4A and ARID4B, interact with the tumor suppressor retinoblastoma protein and are members of the chromatin-remodeling complex. These orthologs function as transcriptional repressors upon recruitment by retinoblastoma protein (Defeo-Jones *et al.* 1991; Lai *et al.* 1999, 2001; Cao *et al.* 2001; Fleischer *et al.* 2003).

Given the putative role of Htk in chromatin modeling, we speculate that it has pleiotropic functions in modulating an astounding array of cellular processes. Since oogenesis in *Drosophila* is an excellent model system to study various aspects of cell and developmental biology, such as cell fate determination, cell differentiation, cell migration, polarization at cellular and tissue levels, inter- and intracellular trafficking, and signal transduction, it was fascinating to explore the pleiotropic functions of *htk* during oogenesis in *Drosophila*.

*Drosophila* oogenesis begins at the anterior tip of the germarium when a germ stem cell divides to form one cell of its own type and the other cystoblast cell (Figure 1D). A cystoblast cell undergoes four synchronous divisions to form a cyst of 16 interconnected cystocyte cells in region 1 of the germarium (Figure 1, C and D) (Spradling 1993; Spradling *et al.* 1997; de Cuevas *et al.* 1997). These cystocytes enter meiosis and initiate the assembly of the synaptonemal complex. Crossing over is initiated in these cells by a controlled induction of double-strand DNA breaks (DSBs) in region 2a, followed by homologous recombination repair. Subsequently, only two pro-oocytes, both of which have four ring canal connections (Figure 1, C and D), proceed to pachytene and generate two meiotic gradients in region 2b (Carpenter 1979; Page and Hawley 2001, 2004; Jang *et al.* 2003). One of the two pro-oocytes is determined as an oocyte and acquires a posterior position in the developing egg chamber in region 3, while other sister cells enter endocycle to form nurse cells. As the oocyte leaves pachytene, the meiotic chromatin releases the components of the synaptonemal complex and condenses into a compact karyosome (King 1970). All DSBs generated in region 2a are repaired through homologous recombination by stage 3. This is followed by the first arrest of oocyte at the diplotene stage of prophase I (Page and Hawley 2001; Resnick *et al.* 2009). Any perturbation in the well-conserved DSB repair machinery initiates the activation of a meiotic checkpoint. Activation of these checkpoints blocks both karyosome formation required for meiotic progression and oocyte polarization (González-Reyes *et al.* 1997; Ghabrial *et al.* 1998, Ghabrial and Schupbach 1999; Morris and Lehmann 1999; Staeva-Vieira *et al.* 2003).

**Figure 1:**
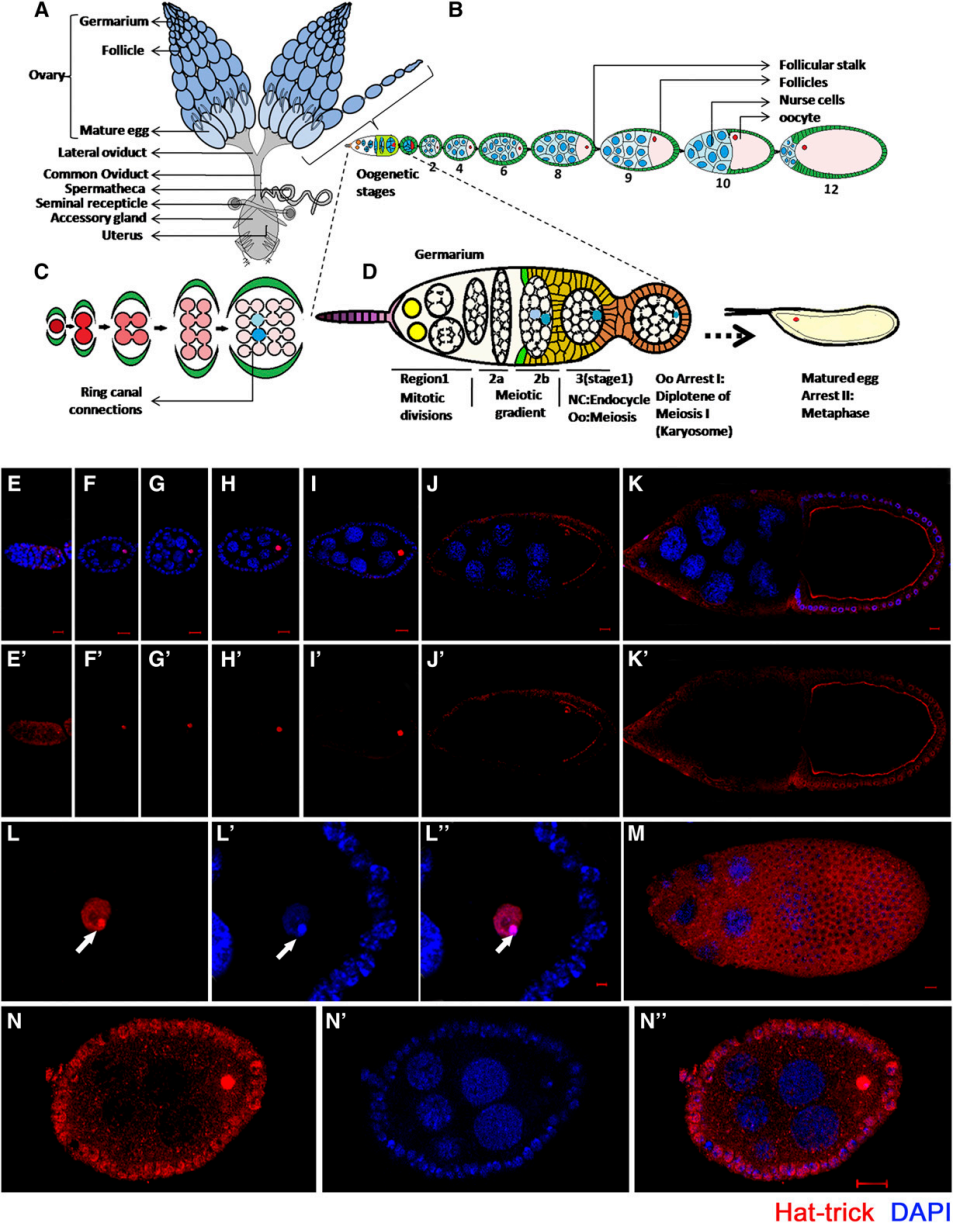
(A–D) Schematic representation of the reproductive system of *Drosophila melanogaster*. (A) Two ovaries consist of ovariole clusters that connect to a common oviduct. (B) An ovariole contains a germarium at the tip followed by sequential stages (S1–S14) of egg chambers. Each egg chamber is composed of an oocyte and 15 sister nurse cells, surrounded by a layer of follicular epithelial cells. (C) Sixteen germ cells, derived from mitotic division of germ stem cells, are interconnected through ring canal connections. Either of the two cells having four ring canals forms the oocyte (blue). (D) Magnified view of germarium. (E–N) Htk protein shows a distinct expression pattern. Confocal micrographs showing the expression pattern of Htk protein during *Drosophila* oogenesis. (E, E′) A cytoplasmic distribution of Htk is observed in all cells of germarium except the nuclear expression (arrow) in the oocyte in region 3. (F–K′) Distribution of Htk (Red) is shown at stage 2 (F, F′), 4 (G, G′), 6 (H, H′), 8 (I, I′), 9 (J, J′), and 10 (K, K′) egg chambers. Htk accumulates in the nuclei of the oocyte until stage 9 egg chamber. At stage 10, Htk localizes at the oocyte cytoplasmic cortex. (L–Lʹʹ) A high-magnification image of an oocyte nucleus at stage 3, illustrating the accumulation of Htk specifically in the karyosome structure, is shown. (L) Note that the bright stain of DAPI marks the highly heterochromatinized region of DNA (Lʹ), DAPI colocalizes with Htk in the karyosome (Lʹʹ) (arrowhead). (M) Upper focal plane of stage 10 egg chamber showing the cytoplasmic expression of Htk in follicle cells. (N–Nʹʹ) To highlight a mild cytoplasmic expression of Htk in follicle cells, the image was overexposed. Scale bar, 10 µm.

Here, we present the multifaceted functions of *htk* during *Drosophila* oogenesis. Htk is expressed throughout oogenesis in different types of cell and is involved in the regulation of various processes during oogenesis. To address the autonomous functions of *htk* in the germline, we generated *htk* germline mosaic clones using the FLP-DFS technique (Chou *et al.* 1993; Chou and Perrimon 1996). This technique combines the germline-dependent dominant female sterile mutation *ovoD1* as a selection for the detection of germline recombination events, and the *FLP-FRT* recombination system to promote site-specific chromosomal exchange. *htk* mutant eggs developed from females carrying germline clones were sterile and had chorion defects. The most frequent chorion defect observed was ventralization of oviposited eggs; this defect was typical of the downregulation of Gurken (Grk)-mediated epidermal growth factor receptor (EGFR) signaling. Similar ventralized eggs were observed in DSB repair mutants. We observed an increased incidence of DSBs, karyosome defects, and altered Vasa in *htk* mutant germaria, which collectively suggest a putative role of *htk* in the early stages of oogenesis. Additionally, we detected defects in oocyte determination, karyosome formation, oocyte positioning, and maturation in *htk* mutant egg chambers. Our study gives an affirmative clue regarding the indispensable role of *htk* during oogenesis in *Drosophila*.

## Materials and Methods

### Fly strains and germline mosaic generation

All fly stocks were maintained on a standard medium of corn meal, yeast, molasses, and agar at 25°. Oregon-R flies were used as wild-type controls. *htk* null mutant alleles—*htk^71^neoFRT19A/FM7*, *htk^39^neoFRT19A/FM7*, and *htk^47^neoFRT19A/FM7*—were kindly provided by Sreedharan *et al.* (2015). *ovoD1hs-FLP neoFRT19A/C*(*1*)*Dx/Y* stock was obtained from Bloomington (BL23880).

For clonal knockdown of *htk* in the germline, we used the FLP-DFS technique (Chou *et al.* 1993; Chou and Perrimon 1996). The *ovoD1hs-FLP neoFRT19A/C*(*1*)*Dx/Y* strain was crossed with *htk^71^neoFRT19A/FM7* to generate germline clones. For control, the *ovoD1hs-FLPneoFRT19A/C*(*1*)*Dx/Y* strain was crossed with *neoFRT19A/FM7*. Heat shock was given to early pupae at 37° for 60 min. Females with appropriate genotype were dissected after 5–10 d for further experiments. In addition, we checked the phenotypes of an experimental cross without giving heat shock and compared them with the phenotypes obtained after heat shock of the same cross. More than 1700 egg chambers and 300 germaria of *ovoD*, *FRT19A/FRT19A* (as a control), and *htk* mutant (experimental) were observed to score the phenotypes.

### Antibody generation and immunostaining of ovary

A unique sequence from the carboxyl terminus (7629–7830 bp) of *Drosophila htk* cDNA (*CG34422* and Gene Bank accession number NM_133102) was cloned into *pGEX-4T-1* vector (GE Healthcare) using restriction sites for *Eco*RI and *Not*I. Next, *Escherichia coli BL21*cells (GE Healthcare) were transformed with the sequence-verified constructs. A single colony of transformants was freshly inoculated in 5 ml of Luria broth (HiMedia Laboratories) with 100 μg/ml ampicillin (Sigma) at 37° on a shaking incubator. For large-scale induction of GST-fusion proteins, 1–2% primary inoculum was added to 100 ml Luria broth with 100 μg/ml ampicillin at 37° on a shaking incubator, and 2 mM isopropyl β-D-1-thiogalactopyranoside (Fermentas) was added when the absorbance of the culture at 600 nm reached 1.00. The lysate fraction was collected after 3 hr of induction in a buffer containing Cell Lytic Express tablets (Sigma) and 1× complete protease inhibitor (Roche). This lysate was mixed with glutathione Sepharose beads (GE Healthcare) to pull down the GST-Htk carboxy-terminal fusion protein. After washing, elution was performed using 15 mM reduced glutathione in 50 mM Tris-Cl. After elution, the purified protein was used for polyclonal antibody generation.

For immunostaining, ovaries were dissected from 5- to 8-days-old flies in cold phosphate buffered saline (PBS). A 1:1 mixture of 3% paraformaldehyde in PBS and heptane was added at room temperature, 25°, for 1 min, followed by 20 min incubation in 3% paraformaldehyde and 5% dimethyl sulfoxide. Tissues were then washed four times in washing solution [a mixture of 1× PBS, 0.2% Triton-X-100, and 0.1% bovine serum albumin (BSA)] for 10 min each. Tissues were incubated in the blocking solution (Tris-PBS with 0.1% BSA and 8% normal goat serum) for 60 min. Primary antibodies were diluted in the blocking solution, added to the ovaries, and incubated overnight at 4°. After four washes in washing solution and incubation in the blocking buffer for 30 min, secondary antibodies were added in 1:200 dilution and incubated for 90 min at room temperature. This was followed by four washes in washing solution for 10 min each. DAPI (4ʹ,6-diamidino-2-phenylindole dihydrochloride) (1 µg/ml) was added for 20 min to the tissues after a PBS wash. After final dissection of each ovariole in cold PBS, samples were mounted in 1,4-diazabicyclo[2.2.2]octane (DABCO). The following primary antibodies were used: polyclonal anti-Htk raised in rabbit (1:100), anti-HP1 raised in mouse (1:100) (Developmental Studies Hybridoma Bank; DSHB), anti-Grk raised in mouse (1:100) (DSHB), anti-Vasa raised in rat (1:100) (DSHB), anti-C(3)G raised in mouse (1:200) (a gift from Prof. R. Scott Hawley), anti-gamma-His2Av raised in rabbit (1:200) (requested from Prof. Kim Stewart McKim), and phalloidin (1:100). The secondary antibodies used were as follows: Alexa Fluor 555 conjugated goat anti-mouse IgG (1:200) (Molecular Probes), Alexa Fluor 488 conjugated goat anti-mouse IgG (1:200) (Molecular Probes), Alexa Fluor 555 conjugated goat anti-rabbit IgG (1:200) (Molecular Probes), and Alexa Fluor 555 conjugated goat anti-rat IgG (1:200) (Molecular Probes).

### Microscopy

Fluorescent images were obtained using a Carl Zeiss LSM780 confocal microscope. Eggshells were visualized by reflected light or dark-field microscopy.

### Western blotting

Ovaries (50) were homogenized in 100 μl of 1× radioimmunoprecipitation assay buffer to prepare crude lysate. Protein lysate was separated on 12% denaturing sodium dodecyl sulfate polyacrylamide gel and transferred onto ImmunoBlot polyvinylidene difluoride membranes (Bio-Rad). Blots were washed with TBST (50mM Tris Base, 150mM NaCl, 0.1% Tween-20), followed by incubation in the blocking buffer (4% skimmed milk in TBST) for 30 min. Blots were probed with mouse anti-Vasa antibody (1:2000) (DSHB). After washing three times in TBST and another blocking for 30 min in the blocking solution, rabbit anti-rat IgG peroxidase conjugated (1:1500) (Bangalore GeNei) was added for 90 min, followed by three washes in TBST. Blots were exposed to a chemiluminescent horseradish peroxidase substrate (Millipore) in a dark room, and signals were documented on X-ray film.

### Data availability

The authors state that all data necessary for confirming the conclusions presented in the article are represented fully within the article.

## Results

### Dynamic expression pattern of Htk

**Figure 2:**
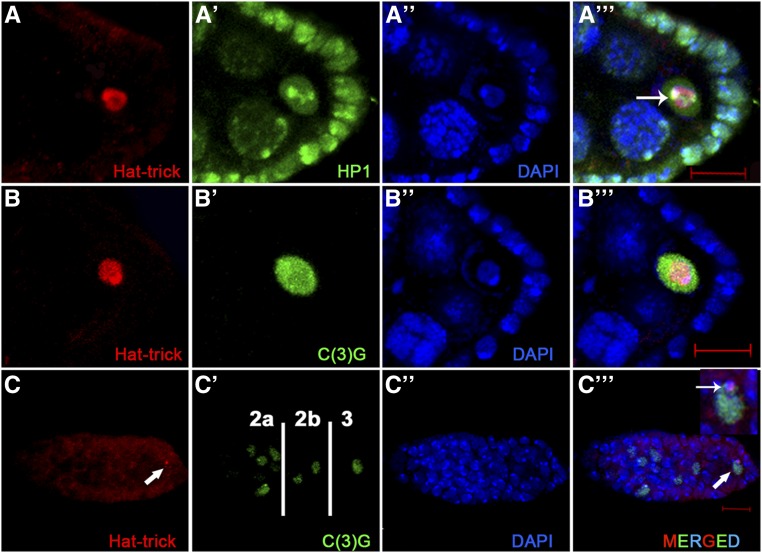
Htk plays a vital part in heterochromatinized karyosome formation and oocyte determination. (A, Cʹʹʹ) Colocalization of Htk with heterochromatin markers within oocyte nucleus is shown by coimmunostaining (arrow in Aʹʹʹ and Cʹʹʹ). Wild-type ovaries were double-stained for Htk (red) and heterochromatin protein 1 (A–Aʹʹʹ), a synaptonemal complex protein C(3)G in a developing egg chamber (B–Bʹʹʹ) and inside a germarium (C–Cʹʹʹ) (green). In regions 2a and 2b of germarium, homogenous Htk expression was present in all germline cells, while in region 3, the Htk protein is predominantly localized within the oocyte nucleus (arrow). Scale bar, 10 µm.

In an attempt to analyze Htk functions in oogenesis, we initiated the assessment of Htk expression in various cell types and stages of progressing egg chambers through immunostaining using a polyclonal anti-Htk antibody. To check the specificity of the anti-Htk antibody, *UAS-HA-htk*, *UAS-GFP* was overexpressed under the control of a *ptc-GAL4* driver, and we observed that the Htk staining and GFP expression overlapped at the anterior–posterior boundary (Patched-domain) of third instar larval wing discs (Supplemental Material, Figure S1, a–d). In addition, Htk expression was completely absent in *htk* null egg chambers (Figure S1, g and h). Together, these results indicate that the anti-Htk antibody was specific. Immunostaining-based experiments using this polyclonal antibody not only indicated the dynamic expression of Htk throughout oogenesis, but also gave an affirmative clue regarding its indispensable role during oogenesis. Htk expression was detected throughout oogenesis, with particularly strong expression from stages 2 to 11. Beginning with the germarium, Htk was expressed in regions 1, 2a, 2b, and 3 (Figure 1, E and Eʹ). The sequential events of development in the germarium include the four mitotic cyst divisions in region 1 trailed by region 2a, where all 16 cells enter the premeiotic S phase, and then a meiotic gradient forms within the two cells with four ring canals in region 2b (Chandley 1966; Carpenter 1979, 1981). Uniformly low levels of Htk expression were observed throughout regions 1, 2a, and 2b of postmitotic cysts. In regions 2a and 2b, homogenous Htk expression was present in all germline cells (Figure 1, E and Eʹ and Figure 2C). The determination of an oocyte occurs during the transition from region 2b to region 3; this transition is followed by the first arrest of the oocyte at diplotene of prophase 1. In region 3, the Htk protein is predominantly localized within the oocyte nucleus (Figure 1, E and Eʹ and Figure 2C). This unique expression pattern signifies its role in oocyte determination and maintenance. From stage 2 onwards until later stages, the Htk protein was localized precisely within the oocyte nucleus (Figure 1, F–J and L), and a mild cytoplasmic expression was observed in the surrounding follicle cells (Figure L–N). We found that Htk expression remained restricted to the oocyte nucleus only until stage 9/10. In subsequent stages, it disappeared from the nucleus and could only be observed along the oocyte cortex (Figure 1K). The cytoplasmic localization of Htk in the oocyte cortex, in later stages of oogenesis, might have a functional implication, which is yet to be determined.

### Htk is crucial for karyosome formation during oocyte arrest

In the *Drosophila* oocyte, after the completion of recombination, meiotic chromosomes cluster together to form a spherical body within the enlarged oocyte nucleus, called the karyosome, which upon maturation facilitates the assembly of spindle microtubules (King 1970). Within the karyosome, the homologous chromosomes are paired at centromeric heterochromatin, and this centromeric heterochromatin of different chromosomes tends to be clustered together (Dernburg *et al.* 1996). The punctate and bright fluorescent point in the karyosome when stained with DAPI indicated the densely heterochromatinized region (Figure 1Lʹ). Htk exhibited a considerable accumulation in the oocyte nucleus, specifically within the karyosome (Figure 1L). The accumulation was relatively high in the heterochromatinized region within the karyosome; accumulated Htk precisely colocalized with the DAPI-stained bright region (Figure 1Lʹʹ).

**Figure 3:**
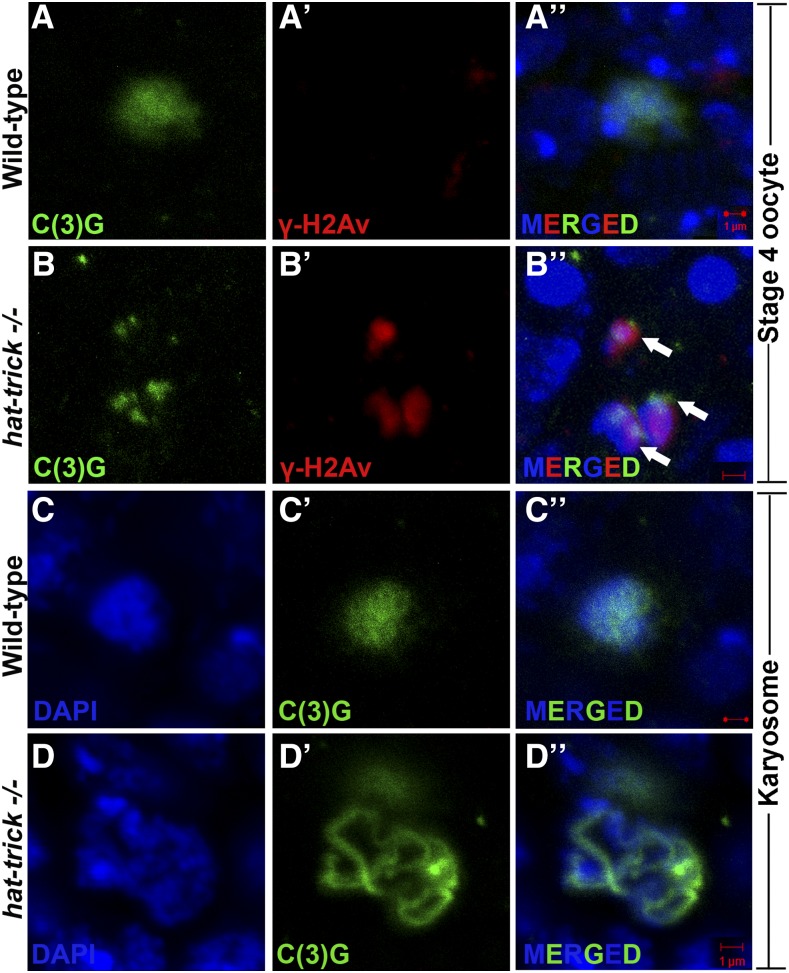
*htk*−/− germaria show karyosome defects. Oocytes are marked by C(3)G (green), and γ-His2Av foci mark DSBs (red). (A–Bʹʹ) Several *htk*−/− egg chambers show fragmented oocyte karyosome as shown by the presence of γ-His2Av foci (red, arrow) beyond germarium stage (here stage 4) (B–Bʹʹ), whereas no γ-His2Av foci are observed beyond wild-type germarium (A–Aʹʹ). (C–Dʹʹ) Decondensed karyosome defects are observed in *htk*−/− egg chambers. It appears diffused (D–Dʹʹ) in comparison with that of the wild type (C–Cʹʹ). Scale bar, 1 µm.

*htk* is predicted to have two splice variants. The longer transcript encodes the chromodomain, an AT-rich ARID domain, RBB1NT (NUC162) domain, and Tudor domain, which are present in the proteins involved in chromatin modeling. These domains endow the protein with chromatin-binding properties. The name ‘Hat-trick’ itself was derived after the putative role of the protein in heterochromatin association (Sreedharan *et al.* 2015). Based on the putative role of Htk in heterochromatin association and our observation of its precise localization within the karyosome, we checked its status with the well-established heterochromatin marker HP1 (Heterochromatin Protein 1) (Klattenhoff *et al.* 2009), which marks all heterochromatic regions within the nucleus of oocyte, nurse cells, and follicle cells. Immunostaining demonstrated that Htk colocalizes with HP1, particularly in the brightly fluorescent sites of the karyosome in the oocyte nucleus (Figure 2, A–Aʹʹʹ). This pattern of immunostaining suggested that Htk might have an association with the heterochromatin clustering and karyosome maintenance. To verify this association, we further analyzed the colocalization of Htk with C(3)G (Crossover suppressor on 3 of Gowen), which encodes transverse filaments of the synaptonemal complex. Within the germarium, the 16 cystocytes formed by four consecutive mitotic divisions are interconnected through ring canals. Two of these cystocyte cells with four ring canals have most of the meiotic features, because they have mature synaptonemal complex as evident from the presence of C(3)G. Eventually, in region 3, the cell-cycle fate of 15 sister nurse cells alters dramatically from the oocyte with strong C(3)G staining (Chandley 1966; Carpenter 1979, 1981; Schmekel *et al.* 1993). In region 3 of the germarium, Htk showed colocalization with C(3)G in the determined oocyte (Figure 2, C–Cʹʹʹ). As soon as the oocyte is determined, it is programmed to enter meiotic arrest. Furthermore, Htk, which was expressed in the cytoplasm of the germarium until region 2, accumulated within the oocyte nucleus in region 3. This colocalization retained until the late stages of oocyte development (Figure 2, B–Bʹʹʹ). We observed that C(3)G and HP1 were present in the interchromatin space and attached to chromatin within the nucleus; however, Htk colocalized only with the chromatin-bound C(3)G and HP1 (Figure 2). The specific expression pattern of Htk in developing egg chambers prompted us to investigate the functions of *htk* in egg chamber development. As homozygous *htk* null flies are lethal, we used the FLP-DFS technique to generate homozygous *htk* mutant clones in the germline (Chou *et al.* 1993; Chou and Perrimon 1996). Three independent *htk* mutant alleles, *htk^39^*, *htk^47^*, and *htk^71^*, were used for germline mosaic analysis (Sreedharan *et al.* 2015). They represent a single lethal complementation group in cytological region 17D6-17F2. Alleles *htk^47^* and *htk^71^* harbor premature stop codons, whereas allele *htk^39^* contains a donor splice site mutation, impairing splicing of intron 2 and likely leading to read-through of the transcript to an intronic premature stop codon (Sreedharan *et al.* 2015). Females that are heterozygous for the autosomal insertion of the dominant female sterile mutation *ovoD1* fail to lay eggs. The egg chambers from the female bearing germline clones devoid of *ovoD1* mutation developed and exhibited different kinds of abnormalities (Chou *et al.* 1993). These different *htk* mutants displayed similar phenotypes; therefore, phenotypes from *htk^71^* mutant allele are shown here. We often observed thread-like or even fragmented karyosomes, which appeared less condensed in *htk*−/− egg chambers (Figure 3), confirming the role of *htk* in karyosome maintenance.

### htk mutant eggs show chorion patterning defects

**Figure 4:**
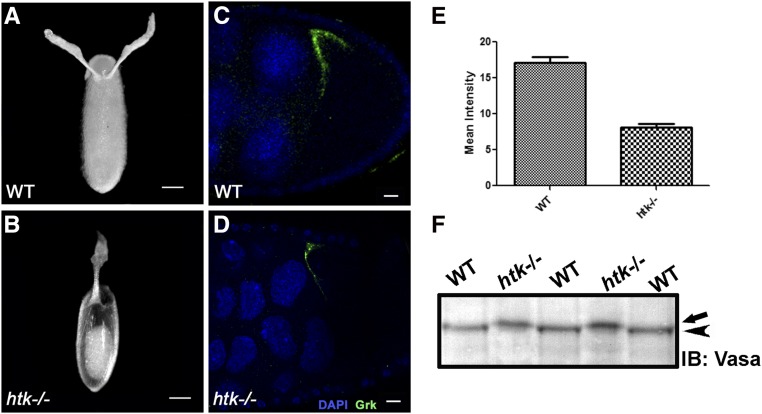
*htk* mutant eggs show ventralization phenotypes, probably owing to reduced levels of Grk protein. Dark-field images of oviposited eggs are shown in the dorsal view. (A) Wild-type (WT) oviposited egg with normal dorsal appendage (DA) morphology. (B) *htk* mutant egg showing ventralization phenotype with single DA. (C and D) Grk protein expression (green) is shown at stage 9 in WT egg chamber (C) and *htk*−/− egg chamber (D). Note that Grk expression is reduced in *htk* mutant egg chambers. (E) Quantification and comparison of mean Grk intensity in equal area of WT and *htk* mutant egg chamber. (F) Western blot showing Vasa protein from WT and *htk* mutant ovarian extract. Vasa from *htk*−/− ovarian extracts (arrow) shows a reduced mobility compared with that from WT extracts (arrowhead). Scale bars, 20 µm (A and B), 2 µm (C and D).

*htk* null eggs exhibited diverse chorion patterning defects (Figure S2j). We observed a wide array of eggshell phenotypes in which the size, shape, and position of dorsal appendages (DA) was altered. There was a gradient in the polarization phenotypes of *htk* null DA. Eggshells were apparently ventralized (Schüpbach 1987; Wasserman and Freeman 1998), with single DA (16%) (Figure 4B) to partially or totally fused DA (Figure S2, a and b) (12%) (*n* = 400 for chorion phenotypes). Only 1.5% of eggshells had three to four DA (Figure S2, d and e). Phenotypes in the DA varied from ventralization portrayed by a single dorsal eggshell filament on the dorsal midline to dorsalization characterized by four DA (Figure 4B and Figure S2e) (Schüpbach 1987). We found that the eggs with single DA formed the majority of the eggs having an altered number of DA. Since reduced Grk expression results in such defects, we further looked at the expression level of Grk in *htk* mutant egg chambers.

### htk is required for proper Grk expression and localization

The follicle cells initiate filament formation according to the dorso-ventral pattern dictated by Grk, a TGF-α-like signaling molecule that has EGF repeat elements (González-Reyes *et al.* 1995; Sapir *et al.* 1998; Peri *et al.* 1999). Dorso-ventral patterning is achieved through the cytoplasmic localization of *grk* mRNA and the regulation of its translation (González-Reyes *et al.* 1995; Roth and Lynch 2009). The variability in patterning defects of *htk* mutant eggs typified the mutations that disrupt the synthesis or distribution of Grk (Schüpbach and Roth 1994; González-Reyes *et al.* 1995; Ghabrial *et al.* 1998). We therefore scrutinized the expression pattern of Grk. *grk* mRNA localizes to the posterior end of the oocyte between the nucleus and cell membrane during early oogenesis. With progression of stage, the oocyte nucleus moves to the anterior dorsal position, where *grk* mRNA becomes localized in a crescent between the oocyte nucleus and cell membrane (Neuman-Silberberg and Schupbach 1993). Grk protein is also restricted to the same region (Figure 4C) and induces a local activation of EGFR in the overlying follicle cells (Neuman-Silberberg and Schupbach 1993, 1994). We observed significant alterations in Grk localization and expression, which were detectable through immunostaining. Interestingly, immunostaining in 15% (*n* = 750 for Grk-stained egg chambers) of *htk*−/− egg chambers displayed significantly reduced levels of Grk expression (Figure 4, C–E). The reduced level of Grk expression might be the cause of ventralized eggs laid by females containing *htk* germline mosaic clones. Additionally, we observed 1.6% egg chambers with ectopic ventral Grk along with dorsal expression, which might be responsible for four DA in the mature egg (Figure S2f), and a few egg chambers where Grk was mislocalized in a ring around the entire anterior side of the oocyte (Figure S2c), presumably leading to a crown of DA material (Figure S2, a and b). Thus, we observed a clear mislocalization of Grk in *htk* egg chambers.

### The htk mutant displays delayed but increased and persistent DSBs

Dorso-ventral patterning defects, similar to those of the mutants of Grk and EGFR-signaling pathway components, were observed in the eggs of females defective for meiotic DSB repair genes, such as okra/dRAD54 (okr), spindle-A/dRAD51 (spn-A), and Cyclin G (CycG) (González-Reyes *et al.* 1997; Ghabrial *et al.* 1998, Ghabrial and Schupbach 1999; Staeva-Vieira *et al.* 2003; Nagel *et al.* 2012). Several studies have linked DSB repair defects and Grk-mediated dorso-ventral defects (Ghabrial *et al.* 1998; Ghabrial and Schupbach 1999; Abdu *et al.* 2002). Persistent DSBs activate the meiotic checkpoint protein Mei-41 (Ghabrial and Schupbach 1999; Abdu *et al.* 2002; Staeva-Vieira *et al.* 2003), leading to the modification of the germline RNA helicase Vasa, which acts as a translation initiation factor for *grk* mRNA (Neuman-Silberberg and Schupbach 1994; Styhler *et al.* 1998; Ghabrial and Schupbach 1999). Misexpressed and mislocalized Grk in turn causes impaired dorso-ventral patterning. Since we observed similar dorso-ventral defects in *htk* null eggs, and *htk* is predicted to be an important component of chromatin modeling, we hypothesized a key role of *htk* in DSB repair.

**Figure 5:**
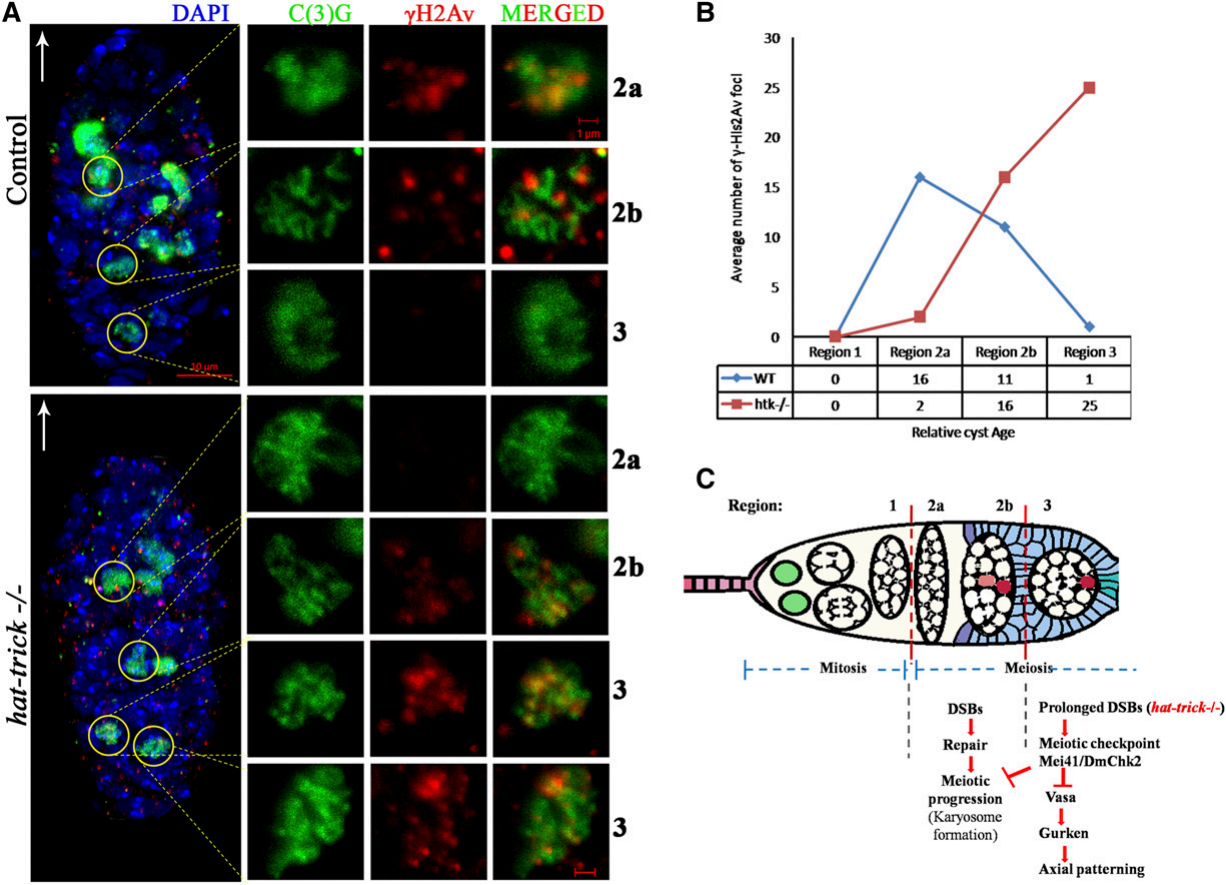
*htk* mutations cause prolonged and increased DSBs. (A) Analysis of γ-His2Av foci in control and *htk* mutant germaria. Anti-γ-His2Av (red) detects DSBs, and anti-C(3)G (green) detects synaptonemal complex. The white arrow points to the anterior tip of the germarium. In wild-type (WT) germaria, most of the γ-His2Av foci were detected in region 2a, with fewer γ-His2Av foci in region 2b and no γ-His2Av foci in region 3. Onset of phosphorylation of γ-His2Av, compared to that in WT flies, was delayed in the *htk* mutants (from region 2b), and the foci persisted until late pachytene stage (in region 3). Two C(3)G positive cells in region 3 of *htk−/−* germaria were observed, which reflected a delay in oocyte determination. Scale bars, 10 µm (whole germarium) and 1 µm (single cell image). (B) Plot showing the average number of γ-His2Av foci as a function of relative cyst age in WT and *htk* mutant germaria. The foci were counted in the pro-oocytes of each of the cysts and normalized. (C) Model for checkpoint-mediated coupling of meiosis to oocyte polarization. Meiotic recombination is initiated by the formation of DSBs in region 2 of the germarium. DSBs are repaired by the proteins of the recombination-repair pathway until region 3. Persistence of DSBs, as in the case of *htk* mutants, probably activates the meiotic checkpoint pathway, resulting in the modification of Vasa, which blocks efficient translation of Grk, leading to axial patterning defects.

To assess the degree of DSBs in *htk* mutant germaria, we carried out immunostaining using an antibody that detects the phosphorylated form of the histone H2A variant (γ-H2Av); this variant accumulates at the sites of DNA breaks and marks meiotic DSBs (Jang *et al.* 2003; Mehrotra and McKim 2006). All the phenotypes observed in the germarium from females containing *htk* germline clones were normalized to those in the germarium of the ovary from *ovoD1*, *FRT19A/FRT19A* females after heat shock. This observation confirmed that the phenotypes in the germarium are specific to *htk* null mutation and not due to *ovoD1* mutation (*Materials and Methods*). In region 2a and 2b of the wild-type germarium, γ-H2Av foci were observed in germ cells, marking the DSBs that were induced to initiate crossing over (Figure 5A). The *htk* null germarium displayed a delayed appearance of γ-H2Av foci (Figure 5, A and B). All DSBs catalyzed within region 2 were repaired until region 3 of the wild-type germarium (Figure 5A) (Jang *et al.* 2003; Mehrotra and McKim 2006). However, the *htk* null germarium displayed delayed repair of DSBs, as evident from the persistence of γ-H2Av staining within pro-oocytes of region 3, which were coimmunostained with pro-oocyte marker anti-C(3)G. We observed an average of 25 γ-H2Av foci in region 3 of *htk*−/− pro-oocytes displaying a defect in DSB repair (Figure 5B). Occasionally, these γ-H2Av foci were observed beyond region 3 of the germarium (Figure 3, B–Bʹʹ).

### htk mutation causes delay in meiotic progression, karyosome defect, and Vasa modification

The increased and persistent presence of DSBs in *htk* null eggs was expected to subsequently activate a Mei-41- and *Drosophila* checkpoint protein 2 (DmChk2)-mediated meiotic checkpoint, as in the case of other meiotic checkpoint mutants (Ghabrial and Schupbach 1999; Abdu *et al.* 2002; Staeva-Vieira *et al.* 2003), which might cause halt or delay the meiotic progression. To confirm that the increased DSBs in *htk* null eggs caused meiotic checkpoint activation, we examined the *htk* null eggs for a block or delay in meiotic progression. One readout for meiotic progression is the restriction of the synaptonemal complex component C(3)G to the oocyte in region 3 of the germarium (Huynh and St Johnston 2000). Meiosis initiates in >1 cell per cyst in region 2 of the wild-type germarium (Carpenter 1979; Page and Hawley 2001). We found that the nuclear C(3)G signal was restricted from the two pro-oocytes to one cell as the cyst attained maturity (Figure 5A). In the *htk*−/− germarium, there was a probable delay in meiosis, which in turn delayed oocyte determination. The delay in oocyte determination was illustrated by two pro-oocytes that exhibited almost equally bright C(3)G staining even in region 3 of the germarium. Notably, penetrance of the two pro-oocytes in region 3 was 58% in *htk* clones containing germarium, whereas it was only 4% in controls (*n* = 348) (Figure 5A). After recombination is over and synaptonemal complex is disassembled, meiotic chromosomes compact into a spherical body called the karyosome (King 1970; Lancaster *et al.* 2010). Activation of the meiotic checkpoint results in diffused, often threadlike, or even fragmented karyosome, which appears less condensed (González-Reyes *et al.* 1997; Ghabrial *et al.* 1998; Ghabrial and Schupbach 1999). These defects were evident in a high frequency in *htk*−/− egg chambers (Figure 3). This observation further confirmed a delay in meiotic restriction and meiotic checkpoint activation. Similar phenotypes have been reported in spindle mutants; these phenotypes have been attributed to delayed oocyte determination (González-Reyes *et al.* 1997).

The meiotic checkpoint activation consequently activates Vasa, a germline-specific ATP-dependent helicase (Ghabrial and Schupbach 1999; Abdu *et al.* 2002; Johnstone and Lasko 2004). We checked the modification status of Vasa in *htk*−/− eggs in order to corroborate whether the effect of DSBs on Grk-dependent dorso-ventral patterning defects is Vasa-mediated. A mobility shift in the western blot, shown by retarded migration of Vasa in *htk*−/− extracts in comparison with that in wild-type extracts, confirmed the posttranslational modification of Vasa (Figure 4F). Vasa is an eIF4A-like translation initiation factor, required for translation of a number of mRNAs, including that of the TGFα-like molecule Grk (Neuman-Silberberg and Schupbach 1994; Styhler *et al.* 1998; Ghabrial and Schupbach 1999). Therefore, we conclude that in the absence of *htk*, the meiotic checkpoint is activated by persistent or increased numbers of DSBs. This activation leads to modification of the germline helicase Vasa that in turn alters translation of *grk* mRNA and impairs establishment of the correct dorso-ventral axis. The Grk-mediated dorso-ventral defects in eggshell phenotype of *htk* mutants are a consequence of meiotic checkpoint activation.

### Other major defects observed in htk mutant egg chambers

**Figure 6:**
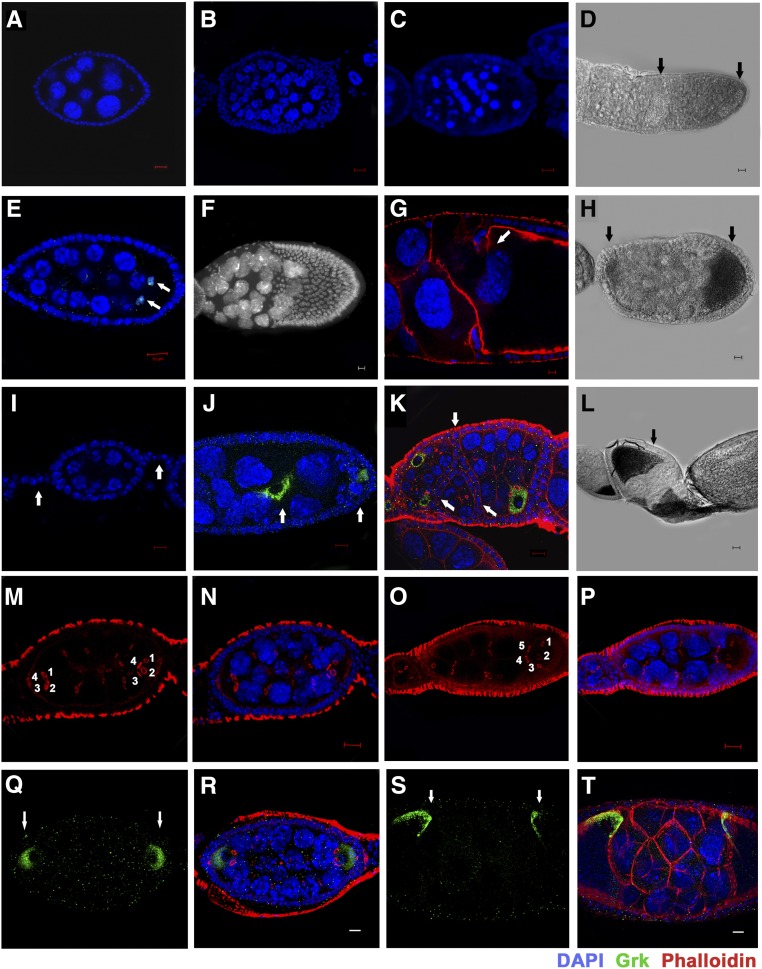
Other major defects observed in *htk* germline clones. (A) A wild-type egg chamber. (B–H and J–L) *htk* −/− egg chambers showed the following phenotypes: almost twice (>30) the normal number of germ cells (B, D, and H), premature apoptosis (C), 14 nurse cells and two oocytes (C3G-positive nuclei) (indicated by the arrow) (E), mispositioned nurse cell nuclei that invaded the oocyte (F), phalloidin staining (red) showing that the nurse cell-oocyte border is disintegrated while the nurse cell nuclei migrated into the oocyte region (arrow) (G), germ cell nuclei of heterogeneous sizes and oocyte having lateral and posterior position as marked by Grk (green) (J), absence of stalk cells resulting in the apposed egg chambers (arrow) (K), egg chamber containing two oocytes at lateral and posterior ends (arrow) (D), anterior and posterior ends (arrow) (H), and oocyte at the anterior end (arrow) (L). (I) A portion of wild-type ovariole showing interconnecting stalk cells (arrow) and egg chamber having posteriorly localized oocyte. Scale bar, 10 µm (except G, 2 µm). (M–T) Cyst encapsulation and germ cell mitosis (cystoblast proliferation) is misregulated in *htk* germline clones. (M and N) *htk*−/− egg chamber containing two vitellogenic oocytes at opposite ends marked by four ring canals (1, 2, 3, 4), which display the multicyst nature of egg chamber. (O and P) An egg chamber consisting of oocyte having five ring canals (1, 2, 3, 4, 5), which illustrated an extra round of mitotic division during cystoblast proliferation. (Q–T) Compound *htk*−/− egg chamber showing ectopic Grk at the anterior and posterior end (arrows) (Q and R), and at dorsal-anterior and dorsal-posterior ends (arrows) (S and T). Scale bar, 10 µm.

In addition to the dorso-ventral polarity and DSB repair defects, a range of other phenotypes were observed in the developing *htk* null egg chambers. Analysis of *htk* mutant ovarioles, using nuclear stain DAPI, revealed ∼25% (*n* = 1750, for egg chamber defects) of the egg chambers with >16 germ cells. These extranumerary germ cell nuclei displayed reduced polyploidy, as evidenced by reduced nuclear size (Figure 6B) in comparison with that of the wild-type egg chamber (Figure 6A). We found that 3.4% of the follicles contained nurse cell nuclei that were heterogeneous in size because of varying degrees of nurse cell polyploidy, implying that cysts of different ages were growing together (Figure 6J). A few *htk*−/− egg chambers displayed 14 nurse cells and two oocytes marked by C(3)G (Figure 6E). These results suggest that mitotic and meiotic cycle control and the switch from mitosis to endoreplication might be misregulated in these cysts. Furthermore, 9.5% of these egg chambers contained pycnotic nurse cell nuclei, irrespective of their early stage, indicative of nuclear fragmentation and early cell death in the nurse cells of the degenerating egg chamber (Figure 6C). We observed extranumerary germ cells containing the egg chamber phenotype up to stage 8/9. This observation confirms that the phenotype is because of *htk* mutation not *ovoD1*mutation, since in *ovoD1/+* females, oogenesis is arrested prior to or at stage 4 (Mével-Ninio *et al.* 1996). Control of germ cell division and positioning of germ cells were found to be impaired in *htk* mutant germline clones. Ring canals are structures that are derived from the contractile rings of cytokinesis during mitosis and give rise to 16 germ cells. These are primarily composed of actin, which can be marked by phalloidin staining (Warn *et al.* 1985; Robinson *et al.* 1994; Mahajan-Miklos and Cooley 1994; Hawkins *et al.* 1996). Interestingly, most of the *htk* null egg chambers with extranumerary germ cells contained two oocytes and copious nurse cells (Figure 6, H, J, and K). This clearly demonstrated the compound nature of the egg chambers, which may arise either owing to an encapsulation defect causing fusion of two cyst cells or because of overproliferation of germ cells. To know the exact cause of the multi-cyst nature, phalloidin staining was performed. Interestingly, it showed various egg chambers with two oocytes (marked by Grk), both having four ring canal connections, suggesting an encapsulation defect as one of the probable causes of the multi-cyst nature of the oocyte (Figure 6, M and N). Grk staining further confirmed that the germ cells, which contained four ring canals and were located at opposite ends, were oocytes (Figure 6, Q–T) and that these egg chambers were compound. Although clones were generated in the germ cells, the defects were observed in the encapsulation of egg chambers by surrounding follicle cells. This may be because of a noncell autonomous effect of *htk* mediated by the cross-talk between germ cells and somatic cells. Moreover, 5% of the egg chambers with extranumerary germ cells contained oocytes exhibiting five ring canals instead of four, indicating an extra round of mitotic division during cyst formation as another cause of extranumerary germ cells in a single egg chamber (Figure 6, O and P). From these observations, we hypothesize that *htk* is also required in the germline for the regulation of mitotic cycles.

In addition, we observed egg chambers in which oocytes were mispositioned, adopting anterior (Figure 6L) or lateral (Figure 6J) positions, signifying an alteration in axial polarity of the egg chamber. Posterior localization of the oocyte depends on the presence of a five- to six-cell-long stalk that links the neighboring egg chambers (Torres *et al.* 2003). There was an absence of interfollicular stalks in *htk* null ovarioles (Figure 6K), in contrast to wild-type ovarioles (Figure 6I). Interestingly, it was frequently observed that the integrity at the boundary between nurse cells and oocyte was lost, and that nurse cell nuclei invaded into the oocyte territory (Figure 6, F and G).

*htk* mutant oviposited eggs displayed various chorion patterning defects. The wild-type oocyte grows in size as a result of cytoplasmic transport or “dumping” of cytoplasmic contents from nurse cells to oocyte as it advances through developmental stages and nurse cells gradually degenerate (Spradling 1993; Cooley and Theurkauf 1994; Robinson and Cooley 1997). By stage 13, nurse cells are completely degenerated after dumping the entire cytoplasmic content and the egg is matured, marked by the presence of DA (Cooley *et al.* 1992). *htk* null egg chambers illustrated incomplete dumping, and there was a delay in the degeneration of nurse cells (Figure S2, h and i). Substantial amounts of cytoplasm were retained within the nurse cells even in the stage-13 egg chamber, possibly because of the collapse of the transport system from nurse cells to oocyte, resulting in a “dumpless” egg chamber phenotype (Figure S2, g–i). The less severe eggs were oviposited, and they were smaller than normal because of a lesser cytoplasmic content. Further, 67% of the oviposited eggs had antler-shaped DA with a broad and thin chorion (Figure S2g), branched or ramified DA (Figure S2h), or a crown of DA material (Figure S2b). This was probably because the extra nurse cell material present in dumpless egg chambers acted as a barrier that inhibited the normal follicle cell migration as they formed the dorsal eggshell filaments (Schüpbach and Wieschaus 1991). We can conclude from these observations that *htk* may regulate a spectrum of vital processes during oogenesis.

## Discussion

### Htk causes prolonged DSBs, which in turn regulate Grk-mediated dorso-ventral patterning

Earlier studies have projected *Drosophila* meiosis as a linear progression of events from synapsis and initiation of DSBs for crossing over to karyosome formation, meiotic arrest, and eggshell patterning. DSB repair is the key checkpoint that bridges meiosis to Grk signaling. Females with mutant DSB repair enzymes lay eggs with dorso-ventral defects known as spindle phenotypes (González-Reyes *et al.* 1997; Ghabrial *et al.* 1998; Morris and Lehmann 1999; Staeva-Vieira *et al.* 2003). Similar to DSB repair mutants, *htk* null eggs exhibited a diverse range of DA-related phenotypes dominated by ventralization defects. We checked the status of each component of the linear progressive events from DSBs levels, meiotic progression, and meiotic checkpoint activation to Vasa modification, Grk expression, and localization, in *htk−/−* eggs, which could cause the axial patterning defects.

Earlier, it was shown that the onset of phosphorylation, in relation to that in wild-type flies, was delayed in DSB repair-defective mutants such as *spn-A*, *spn-B*, and *spn-D* (paralog to *Rad51*) and *okr* (paralog to *Rad54*) mutants. Furthermore, the foci persisted in the late pachytene stage in these mutants (Mehrotra and McKim 2006). We speculate a very similar situation for *htk* mutants, which also showed a delayed appearance of DSB formation (Figure 5, A and B) indicating that *htk* had an important function in DSB formation or γ-H2Av phosphorylation. All DSBs catalyzed within region 2 are repaired until region 3 of the wild-type germarium. Our results have demonstrated increased and persistent DSBs in *htk* mutant eggs, as evident by accumulated and persistent γ-H2Av foci present up to region 3 of the germarium or later stages. The *htk* null germarium displayed delayed repair of DSBs similar to that in the case of DSB repair-defective mutants. To determine whether the increased DSBs in *htk* null eggs activates a meiotic checkpoint, we analyzed *htk* null eggs for any block or delay in meiosis progression. In the context of meiotic progression, there are reports on the restriction of C(3)G to the oocyte in region 3 of the germarium (Huynh and St Johnston 2000). C(3)G staining in the *htk* null germarium showed the presence of two C(3)G-positive pro-oocytes in region 3 of the germarium, which reflects a definite delay in meiosis. Another outcome of the meiotic checkpoint activation and the resulting delay in meiotic progression is a diffused and thread-like morphology of karyosomes, which was observed frequently in *htk* mutants.

The downstream effect of meiotic checkpoint activation is modification of Vasa, a germline-specific ATP-dependent helicase. Our results have demonstrated that the increased and persistent DSBs in *htk* mutant eggs circuitously cause posttranslational modification of Vasa, possibly by activating Mei-41 and DmChk2, as reported earlier for other meiotic double-strand repair mutants (Ghabrial *et al.* 1998, Ghabrial and Schupbach 1999; González-Reyes *et al.* 1997; Staeva-Vieira *et al.* 2003; Nagel *et al.* 2012). Since Vasa plays an important part in the translation of several mRNAs, including that of *grk*, its modification affects the localization and translation of Grk in *htk* mutant egg chambers. Misregulated and mislocalized Grk led to dorso-ventral patterning defects in *htk* mutant eggs, as evidenced by the appearance of eggs with axial patterning defects.

In summary, *htk* mutations increased the incidence of DSBs, delayed DSB repair, delayed meiotic restriction, affected karyosome morphology, and altered Vasa mobility and consequently mislocalization of Grk in *htk* mutant egg chambers (Figure 5C). These observations clearly suggest the involvement of Htk in an early stage of meiotic recombination repair during oogenesis.

### Multifaceted role of Htk during oogenesis

The essential role of chromatin modeling proteins during oogenesis has become evident in recent years. Emerging data indicate the significance of proteins containing the chromodomain (*e.g.*, Rhino, HP1), ARID domain (*e.g.*, Osa), and Tudor domain (*e.g.*, Vasa), in oogenesis (Styhler *et al.* 1998; Tomancak *et al.* 1998; Vazquez *et al.* 1999; Volpe *et al.* 2001; Carrera *et al.* 2008; He *et al.* 2014). Htk is predicted to harbor these domains and shows a unique expression pattern during oocyte development in *Drosophila*. The presence of these domains in Htk protein and its nuclear and cytoplasmic localization reflect its functions in the nucleus and cytoplasm. Expression studies, as well as mutant analyses, have revealed the involvement of Htk in a multitude of events throughout oogenesis. The restriction of Htk specifically within the karyosome since its formation and its colocalization with the heterochromatin markers has validated its role in karyosome formation and maintenance. This association was further confirmed by the observation of thread-like diffused karyosomes in *htk* mutant clones in the germline.

Germline mosaic analysis has shown that *htk* is required for maintaining proper germ cell number, nurse cell–oocyte border, oocyte determination, germ cell positioning, and cyst encapsulation inside the germarium. In the germline, we found that *htk* was involved in the control of mitotic cycles during cyst formation, in the regulation of nurse cell endoreplication, and in nurse cell dumping. Further, *htk* mutant clones in the germline show impaired morphology and distribution of the germarium, delay in oocyte determination, and mislocalized oocytes.

The chromatin binding and modeling property of Htk protein accounts for its speculated role in regulating a wide range of cellular processes in a direct or indirect fashion. Being a chromatin-binding protein, Htk can regulate the expression of a spectrum of genes, which in turn might affect an array of independent processes. Since it is a novel chromodomain protein, the wide array of genes regulated by Htk is yet to be explored. Recently, it has been shown that *htk* mutation results in decreased TDP-43 protein expression without any significant change in the TDP-43 transcript levels; this decrease in TDP-43 protein could be the reason for the suppression of motor neuron degeneration associated with ALS (Sreedharan *et al.* 2015). This ability of chromodomain proteins to regulate various other genes is responsible for multiple functions of *htk* during oogenesis. We have also confirmed that when females bearing *htk* mutant germline clones were crossed with wild-type (*htk* +/+) males, embryos did not hatch or develop. We may conclude from germline mosaic analysis that *htk* is required for maintaining female fertility and that *htk* mutant eggs are sterile. Other than oogenesis, Htk may have pleiotropic functions that are yet to be explored in regulating different aspects of many other developmental and cellular processes.

## Supplementary Material

Supplemental material is available online at www.g3journal.org/lookup/suppl/doi:10.1534/g3.117.300526/-/DC1.
