# Supplementary material for: Pleiotropic Functions of the Chromodomain-Containing Protein Hat-trick During Oogenesis in Drosophila melanogaster
Source: G3 (Bethesda). 2018 Jan 24;8(3):1067–77. doi: 10.1534/g3.117.300526 (PMC5844294; doi:10.1534/g3.117.300526)
Supplement: Supplementary file 1 [file 1067FigureS1.docx]

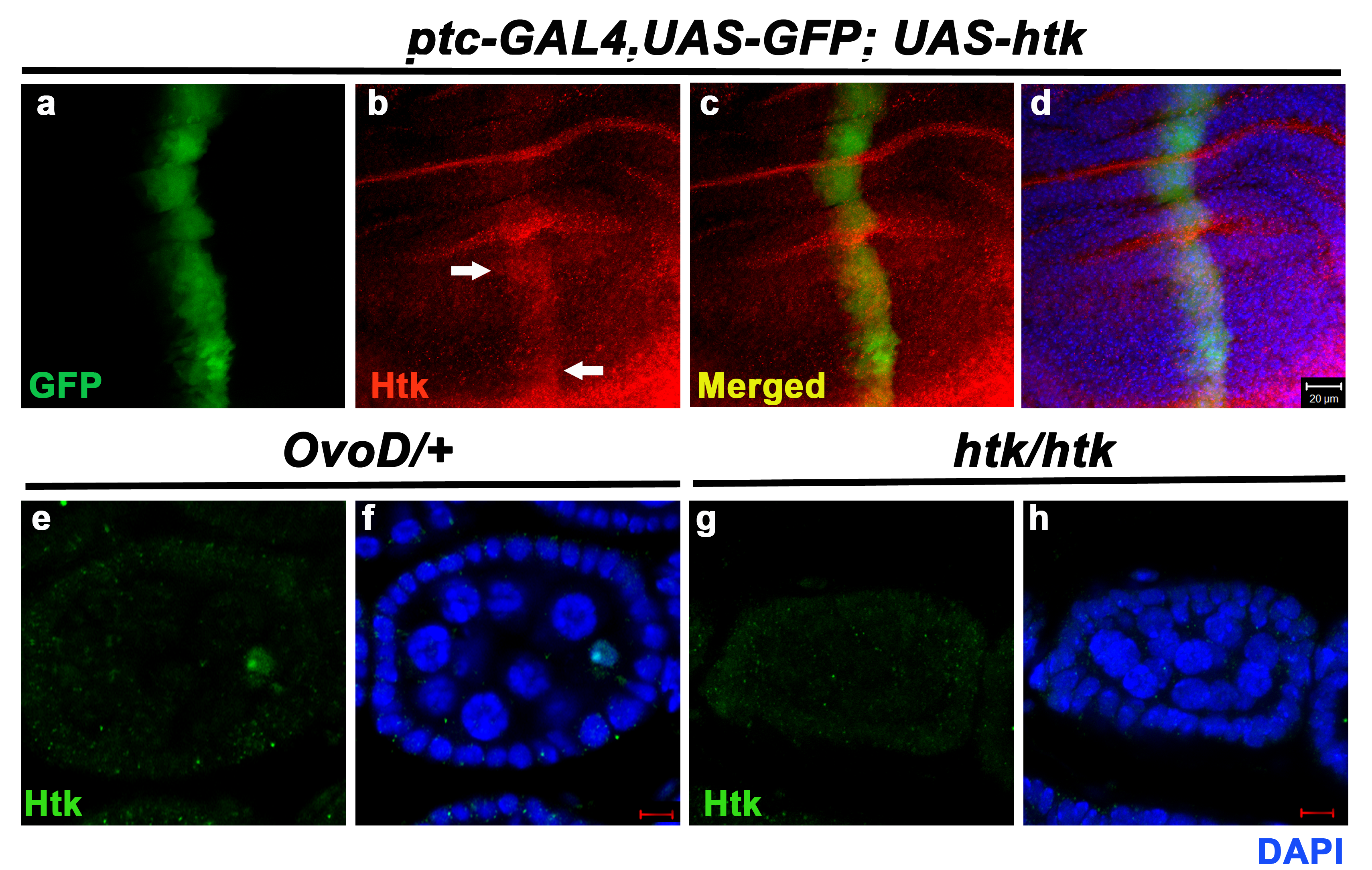


**Figure S1. Analysis of the speciﬁcity of the rabbit anti-Htk antibody. a-d.** Htk was ectopically overexpressed at the anterior-posterior (A-P) boundary region [marked by GFP, (a)] of wing disc using ptc-GAL4 driver. (b-d) Anti-Htk antibody detects both ectopically overexpressed Htk at A-P boundary (marked by arrow) as well as wild type Htk. **e-h.** Anti-Htk antibody detects wild type Htk protein in the nucleus of control oocyte (e-f), but no Htk protein was observed in the projection image of the oocyte of *htk-/-* egg chamber (g-h). Scale bars, 20 µm (a-d), 10 µm (e-h).
