# Supplementary material for: Pleiotropic Functions of the Chromodomain-Containing Protein Hat-trick During Oogenesis in Drosophila melanogaster
Source: G3 (Bethesda). 2018 Jan 24;8(3):1067–77. doi: 10.1534/g3.117.300526 (PMC5844294; doi:10.1534/g3.117.300526)
Supplement: Supplementary file 3 [file 1067FileS1.docx]

**Figure S1. Analysis of the speciﬁcity of the rabbit anti-Htk antibody. a-d.** Htk was ectopically overexpressed at the anterior-posterior (A-P) boundary region [marked by GFP, (a)] of wing disc using ptc-GAL4 driver. (b-d) Anti-Htk antibody detects both ectopically overexpressed Htk at A-P boundary (marked by arrow) as well as wild type Htk. **e-h.** Anti-Htk antibody detects wild type Htk protein in the nucleus of control oocyte (e-f), but no Htk protein was observed in the projection image of the oocyte of *htk-/-* egg chamber (g-h). Scale bars, 20 µm (a-d), 10 µm (e-h).

**Figure S2. Htk affects chorion patterning.** Dark field images of oviposited eggs are shown in the dorsal view. **a.** Partially fused dorsal appendages **b.** Completely fused dorsal appendages resulting into a crown-shaped dorsal appendage, probably because of a ring-shaped Grk at the anterior end (arrows) **(c)** in comparison to the crescent-shaped wild type Grk **(c’**) (arrow). **d-e**. Dorsalization phenotype was shown by ectopic DA with 3 DA (d) and 4 DA (e). The observed dorsalization phenotype may arise due to ectopically expressed Grk at ventral side (arrow) (**f**) of oocyte in *htk*-/- egg chamber in comparison to that in the control (f’). **g-i**. Failure of rapid cytoplasmic transport from nurse cell to oocyte was observed in *htk*-/- egg chambers. Please note the large fraction of the cytoplasm retained in nurse cells. Dorsal ﬁlaments are formed on the top of the remaining nurse cells. Such DAs were broad, branched, and tapering at the ends. These appendages were thicker or occasionally shorter than wild type and sometimes had extra material. **j.** A graph representing percentage of different types of dorsal appendage-related phenotypes. Scale bars, 20µm (a, b, d, e, g, i), 10 µm (c, c’, f, f’).
